# Influencing factors of sports tourism safety accidents in Tibet, China: fsQCA analysis based on the SCM

**DOI:** 10.1371/journal.pone.0334226

**Published:** 2025-10-27

**Authors:** Kejun Wu, Aoxue Xing, Jingbo Zhou, Lihui Su, Sen Zhang, Shuanyan Yang

**Affiliations:** 1 Tourism College of Northwest Normal University, Lanzhou, China; 2 Tourism Development Research Academy of Gansu, Lanzhou, China; Hunan University, CHINA

## Abstract

A comprehensive analysis of the systemic causes of safety accidents in sports tourism on the Qinghai-Tibet Plateau is significant for high-quality development. Utilizing 32 verified accident cases (2010–2025) in the Tibet Autonomous Region of China, this study extracted six critical factors through content analysis: organizational professionalism, rescue capacity, management systems, natural environment, tourist vulnerability, and tourist behavior. The Swiss Cheese Model (SCM) analyzed latent/active failures through case reports and regional environmental data, while the fuzzy-set Qualitative Comparative Analysis (fsQCA) deciphered nonlinear configuration paths across six factors. The results reveal that sports tourism accidents in Tibet arise from the coupling of multiple factors. Specifically, the combination of six influencing factors constitutes the causal paths for severe and general accidents. Among these factors, environmental factors and tourist characteristics are key contributors to accidents. Based on these findings, it is essential to establish a sports tourism risk prevention system for Tibet, which should comprise four layers: natural defense, behavioral defense, managerial defense, and rescue defense. This study deepens the understanding of sports tourism safety accidents on the Qinghai-Tibet Plateau. It integrates the SCM and the fsQCA method, contributing to sports tourism safety research. The proposed risk prevention system provides useful references for local safety management. Future research can focus on the dynamic changes of influencing factors and test the research framework and risk prevention system’s applicability in other similar areas.

## 1. Introduction

The development of sports tourism in China is still in its infancy. National laws and regulations, regulatory policies, industry standards and public safety awareness lag behind the rapid development of the industry. This gap is particularly evident in ecologically fragile and high-risk environments such as the Tibetan Plateau, where tourism safety faces severe challenges and fatal accidents occur frequently. The extreme geographical and climatic conditions in Tibet, including an altitude of more than 4000 meters, unpredictable snowstorms, an anoxic environment and treacherous terrain, have multiplied the risks of sports tourism [1]. Studies show that compared with low-altitude tourism activities, the probability of life-threatening events (such as acute altitude sickness, hypothermia and fatal falls) occurring in high-altitude destinations is 30–50% higher [2]. Despite these documented dangers, the allure of adventure continues to draw an increasing number of thrill-seekers. The consequences of tourism accidents in such special environments go beyond direct casualties and property losses, often triggering large-scale rescue operations, ecological degradation and significant social backlash. In view of the obvious dangers of plateau sports tourism, coupled with its unsustainable growth trajectory, the study of safety accidents in Tibet is of great significance for the sustainable development of sports tourism in Tibet, the ecological safety of tourism [3], and the safety of tourism in high altitude or extreme environments.

In the past five years, the number of literatures based on sports tourism safety accidents has increased sharply, gradually becoming a hot issue in tourism risk research. Most studies are centered on the tourists themselves, analyzing the causes of risky behaviors of sports tourism tourists [4]. Other scholars, integrating knowledge from disciplines such as medicine, kinesiology, and geography, analyzed the influence of factors like the natural environment, management systems, and tourist behavior on a certain sports activity, and gradually began to explore the multiple causes of accidents [5–8]. Traditional descriptive statistics or linear regression methods failed to capture the complex and nonlinear interactions among influencing factors [9]. Recent research has highlighted the need for a systematic approach to understanding sports tourism accidents. By combining qualitative and quantitative methods, an in-depth analysis was conducted on the causes and structure of the accident. It is necessary to utilize techniques from mathematics, computer science and psychology to analyze the special risk factors of sports tourism projects and identify risk information from the causes of accidents [10–12]. However, a systematic analytical method has yet to be formed. Qualitative comparative analysis (QCA), especially fuzzy set QCA (fsQCA), provides a robust alternative by studying how the combination of conditions leads to results [13]. This method has been effectively applied in safety analyses, including engineering, public health, transportation and business [14–17]. Meanwhile, the SCM provides a theoretical framework to categorize accidents into latent failures and active failures [18]. Integrating these approaches allows for a holistic investigation of sports tourism accidents, addressing gaps in existing research.

At present, most studies focus on the distribution characteristics of safety accidents and single influencing factors, while research on the coupling effect of multiple influencing factors is relatively scarce. And these studies usually ignore the tourists’ own conditions and behaviors, as well as external influencing factors such as the social environment. From the perspective of research fields, there are relatively few studies on tourism safety in plateau areas or extreme environmental tourism zones. Therefore, it is of great significance to identify multiple influencing factors of sports tourism safety accidents in plateau areas and analyze their interactions from the perspective of configuration.

**Fig 1:**
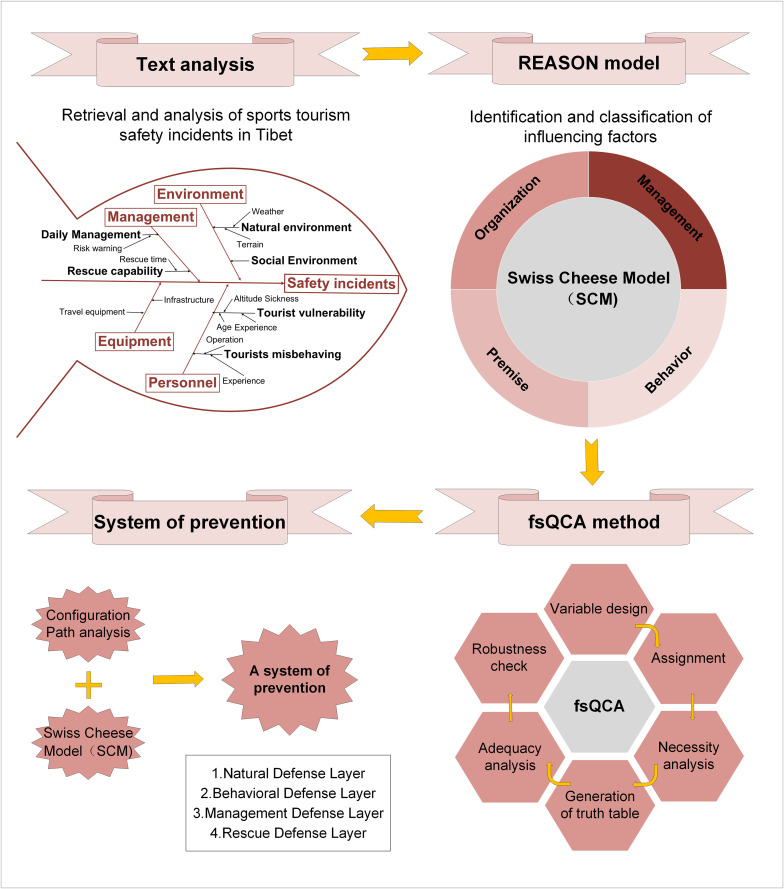
The integrative SCM - fsQCA framework diagram of this study.

This study, by analyzing 32 sports tourism accidents from 2010 to 2025, answered the following questions: (1) What are the spatial-temporal characteristics of sports tourism safety accidents in Tibet? (2) What are the main factors affecting sports tourism accidents in this area? (3) Which configuration paths will cause serious accidents and secondary accidents? The schematic diagram of the integrated SCM-fsQCA framework in this study illustrates the methodological approach adopted (Fig 1). Before writing the article, use the text analysis method and fishbone diagram method to initially identify the influencing factors in the case. Subsequently, based on the SCM model, these factors are classified into four distinct levels: organization, management, environment, and behavior. Then, fsQCA was used to investigate the configuration paths that led to the accident, clarifying how these factors interact to cause serious or minor accidents. Ultimately, based on the analysis results, develop a four-layer risk prevention system that includes natural, behavioral, management and rescue dimensions.

## 2. Materials and methods

### 2.1. Study area

The Tibet Autonomous Region, situated in the southwest of Qinghai-Tibetan Plateau between 26°50’-36°53’N and 78°25’-99°06’E, covers an area of approximately 1,202,800 km^2^ [19]. It includes six prefecture-level cities and one autonomous region, accounting for more than half of the total area of the Qinghai-Tibet Plateau. About 85.1% of Tibet’s altitude is above 4,000 meters, earning it the titles “Roof of the World” and “Third Pole of the Earth” [20]. It is one of the highest and most geographically complex regions in the world. Tibet boasts world-class sports tourism resources such as Mount Everest, Namtso Lake, and Mount Kailash, attracting global adventure and outdoor sports enthusiasts. In recent years, the local government has actively promoted the integration of tourism with sports and culture, developing high-altitude mountaineering, glacier hiking, and off-road cycling projects. Batsong Tso Scenic Area entered the “Top 10 Sports Tourism Destinations in China for 2020” thanks to its globally famous mountain biking, hiking and camping programs [21]. In 2024, Tibet’s sports tourism income exceeded 3.5 billion yuan, reflecting a growth rate of 6.2% [20].

### 2.2. Data collection

To ensure consistency in data selection, this study defines sports tourism based on relevant literature, describing it as an activity in which tourists engage in or observe various sports events during their travel, using the various outdoor sports activities and facilities available at the destination. This includes various adventure activities, recreational sports, and off-road cycling, etc.

After defining sports tourism, this study focused on the period from January 1, 2010, to March 1, 2025, and used 7 keywords such as “hiking” “crossing” “mountaineering” and “cycling” along with terms like “safety” “accident” “fall” “injury” and “death.” These were combined and searched on the internet. The selected sports tourism accident cases were all sourced from news reports by mainstream media outlets, including The Paper, NetEase News, and Sina News, with each case appearing on at least three major websites. A total of 37 cases were collected.

The criteria for selecting fsQCA analysis cases are: first, the cause, process, and outcome of the event must be clearly defined, with detailed descriptions that are suitable for coding; second, the event’s characteristics should be diverse, with the occurrence stemming from different stages, forms, and attributes to ensure the diversity and completeness of the cases [22]. Based on these criteria, cases with incomplete or disputed information were excluded, resulting in 32 typical cases for final analysis.

### 2.3. Methods

The SCM and fsQCA used in this article are complementary at the methodological level in a “vertical layering - horizontal configuration” manner. SCM can view system security as multiple layers of defense and consider risk factors as security vulnerabilities on the defense layers. By locating variables to different levels, SCM can systematically reposition the causal variables in unstructured text, providing a theoretical anchor point for the subsequent selection and calibration of fsQCA condition variables. Provide a structured accident cause-level framework for fsQCA, endowing configuration analysis with theoretical depth. fsQCA examines how security vulnerabilities between levels are aligned and materializes them into quantifiable configuration paths. Meanwhile, the conformance – coverage metric of fsQCA can guide the priority of vulnerability fixes. In short, SCM answers the question of “What are the unsafe factors?”, while fsQCA answers the question of “Which combinations of unsafe factors will lead to safety accidents?” The two work together to complete the closed loop from theoretical layering to empirical configuration.

#### 2.3.1. Swiss cheese model.

The SCM is a complex accident causation model proposed by British psychologist James Reason in 1990, also known as the “REASON model” or “Aviation accident theory model”. The model was first applied to high-risk industries such as medical, transportation and mining [23–25]. Now it is extended to risk management and accident analysis in industries such as sociology and food safety, the mechanism of the accident is revealed and the complexity of the accident is explained [26,27]. Most scholars use the content of the model to systematically analyze all aspects of the accident, identify the potential risk factors that may lead to the accident, summarize the risk, and evaluate the overall risk level. In addition, according to the multi-layer defense mechanism proposed by the model, some scholars study the prevention mechanism and prevention strategy of the accident.

In recent years, many scholars have tried to improve and expand the SCM, and improve the applicability of the model. When Chinese scholars introduced the SCM, they used different analysis methods and made localized improvements based on China’s actual situation, the SCM is applied to Air Force flight training and safety accidents in specific industries in China [28,29]. Foreign scholars have extended the SCM in combination with other theories. Seshia et al. proposed a method of “Closing the Swiss cheese loophole” to improve patient safety [30]. Akuh and Atombo applied the SCM to the analysis of road transportation accidents and proposed an accident analysis method based on system theory [31]. Torres et al. combined the ACCIMAP and STAMP methods with the SCM, and proposed a new method for analyzing road transportation accidents based on the system theory, for the analysis of human errors in complex manual assemblies, the importance of dynamical system analysis is emphasized [32]. In summary, the SCM has been widely used in the study of safety accidents, but there are relatively few literatures on the analysis of tourism safety accidents.

In this study, the SCM serves as the theoretical basis for analyzing the configurational factors affecting sports tourism safety accidents in Tibet. This is reflected in three key aspects: first, the SCM emphasizes that accidents are the result of “systemic holes” created by multiple layers of defense, which is highly relevant to the multidimensional risk sources of high altitudes, extreme climates, and complex terrains in Tibet. Secondly, sports tourism activities usually require professional knowledge and skills, participants face high risks, and these activities often have strong time sensitivity and social influence, necessitates an analysis of the non-linear interactions between defense layers [33]. The “hole alignment” mechanism of the SCM reveals the nonlinear accumulation effect of defense layer failure, thus analyzing the interaction between different defense layers, rather than the linear effect of a single condition. Thirdly, sports tourism involves a wide range of factors, including natural, human, and technical risks, making the SCM highly applicable to the analysis of the influencing factors of sports tourism safety accidents in Tibet [34].

#### 2.3.2 fsQCA method.

The Qualitative Comparative Analysis (QCA) method was proposed by Charles C. Ragin in 1987 [13]. Fuzzy-set QCA (fsQCA) is a case-oriented research method within QCA, based on Boolean algebra and set theory to examine the relationship between antecedent conditions and outcomes [35].

Compared with the traditional Quantitative analysis method, QCA method can capture the nonlinear relationship between variables and multiple concurrent causal effects, which is suitable for dealing with complex social phenomena [36]. It’s a method that facilitates the understanding of complex situations involving multiple factors potentially contributing to an outcome [37]. Since the 21 st century, fsQCA has been widely used in safety accident analysis, especially in the fields of medicine, transportation, commerce and engineering [38–41].

In terms of content, it focuses on the analysis of the complex causal relationship between the accident and various factors, reveals the path and mechanism of the accident, and points out the key causes of the accident. The outbreak mechanism and governance strategy of coal mine safety accidents, the occurrence mechanism of construction safety accidents, the risk of traffic operation, public opinion and public health events are the focus of scholars’ research [16,42–45]. In contrast, the application of fsQCA in the field of analyzing tourism accidents started late, and the number of literatures is not large, which indicates that fsQCA can be used to analyze tourism accidents. The team from the School of Tourism, China Huaqiao University, used fsQCA to analyze the safety accidents of various types of tourism, such as ice and snow tourism, outdoor sports, and night tourism [46–48]. It is an important institution for the study of tourism safety accident configuration causes, and has certain influence. Methodologically, scholars have combined fsQCA with methods such as PLS-SEM, Bayesian Network, and Meta-analysis, in conjunction with relevant theoretical frameworks from other disciplines [49–51]. However, the number of literatures combined with other methods is still small and still in the exploratory stage.

This study chose fsQCA to analyze the configurational characteristics of safety accidents in Tibet’s sports tourism for three reasons: first, the factors influencing sports tourism safety accidents are complex, and fsQCA excels in analyzing multiple, concurrent causal relationships; second, fsQCA is suitable for analyzing smaller sample sizes, which aligns with the limited number of cases available for Tibet’s sports tourism safety accidents; third, the use of SCM to categorize safety factors makes fsQCA an appropriate tool for analyzing the complex interactions of multiple conditions contributing to accidents.

## 3. Result and analysis

### 3.1. Descriptive statistics

#### 3.1.1. Type distribution characteristics.

From 2010 to 2025, the types of sports tourism safety accidents in Tibet mainly include seven categories: camping, cycling, self-driving, hiking, climbing, cruise ships and adventure (Table 1). Among them, hiking and cycling accidents are significantly more common than other types. This might be due to the fact that routes such as the Sichuan-Tibet Line, the Yunnan-Tibet Line, the Qinghai-Tibet Line, and the Xinjiang-Tibet Line are increasingly attracting the attention of sports tourism tourists. In addition, compared with others, these two types of sports tourism activities have higher requirements for tourists’ physical fitness and a greater risk of safety accidents in high-altitude areas.

**Table 1:** Distribution of types of sports tourism safety accidents in Tibet (2010-2025).

Type	Number	Proportion(%)
Camping	1	3.1
Cycling	9	28.1
Self-driving	1	3.1
Hiking	10	31.3
Climbing	6	18.8
Cruise ship	1	3.1
Adventure	4	12.5

#### 3.1.2. Spatio-temporal distribution characteristics.

**Fig 2:**
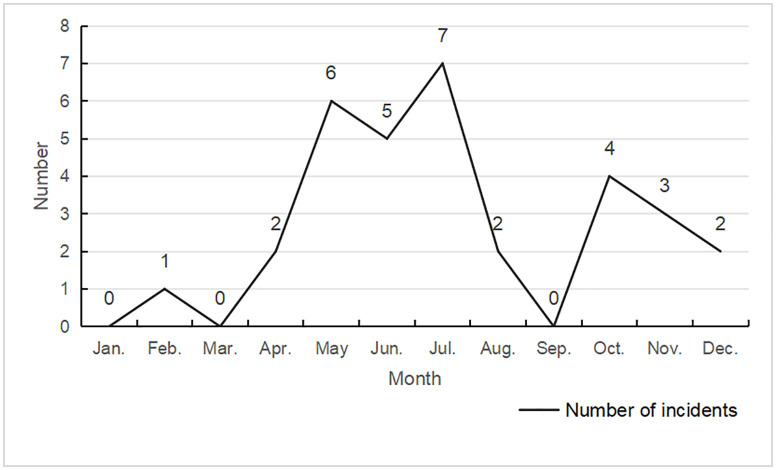
Monthly distribution map of sports tourism safety incidents in the Tibet Autonomous Region (2010-2025).

The temporal distribution of sports tourism safety accidents in Tibet is shown in the Fig 2. Overall, the occurrence time of sports tourism safety accidents in Tibet varies significantly by season. The number of accidents is the highest in July, with only three months having no accidents. This indicates that sports tourism has gradually become a part of People’s Daily lives, not just limited to the peak tourist season. Sports tourism safety accidents in Tibet mainly occur from May to July. This is mainly because during this period, the number of tourists is relatively large and the base of tourists is large, making safety incidents prone to occur [52].

**Fig 3:**
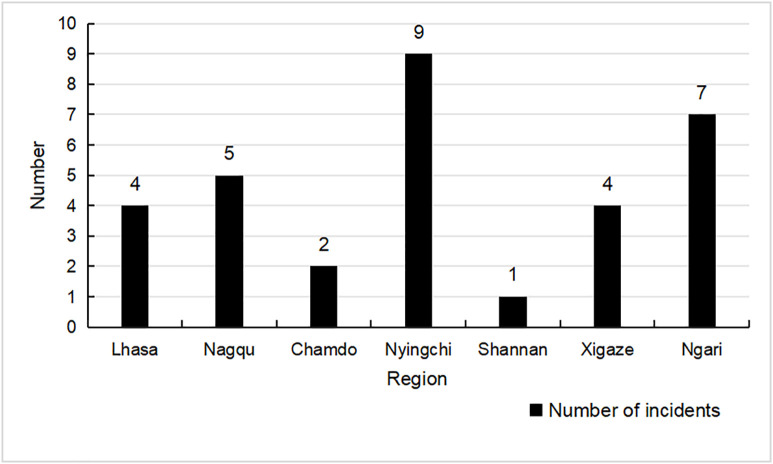
Distribution map of areas where sports tourism safety incidents occurred in the Tibet Autonomous Region (2010-2025).

The spatial distribution of sports tourism safety accidents in Tibet from 2010 to 2025 is shown in Fig 3. Overall, accidents have occurred in all prefecture-level cities and regions, indicating that sports tourism safety accidents in Tibet have shown a trend of frequent occurrence in many places. Among them, the accident distribution in Nyingchi City and Ngari Prefecture is relatively concentrated. This is mainly because these two places are the main destinations for hiking and cycling tourism in Tibet, such as “Tongmai Natural Danger”, Mount Kailash, and Pangong Lake, where the number of tourists is larger and the accident rate is higher. This conclusion corroborates the one drawn in the type distribution characteristics mentioned earlier.

### 3.2. Identification and classification of influencing factors

#### 3.2.1. Identification of influencing factors.

In order to comprehensively extract the causative variables of sports tourism safety accidents in Tibet, this study used the fishbone diagram method to conduct open-ended factor identification of sports tourism accident reports in Tibet. Starting from the particularity of tourist destinations and tourism activities, this article focuses the core of identifying influencing factors on high-risk tourism activities in extreme environments. Sort out the problems according to “people, events, time, environment and objects”, and determine the four core factors of personal factors, environmental factors, equipment factors and management factors as the main framework for analysis. Then, read the accident report sentence by sentence, extract all the phrases directly related to the accident. In the process of identifying influencing factors through accident reports, inter-coder checks were conducted. Finally we obtain 17 high-reliability factors to form the original entry pool (Table 2) for subsequent classification.

**Table 2:** Pool of influencing factors.

Core Factors	High-reliability Factors
Personal Factors	1. Physical or mental fitness of visitors 2. Tourist experience and skills 3. Altitude Sickness 4. Accident due to improper operation 5. Taking risks 6. Low safety awareness decision-making
Environmental Factors	1. Atmospheric Environmental Risk 2. Geo-environmental risks 3. Natural disasters
Equipment Factors	1. Inadequate infrastructure 2. Tourist readiness
Management Factors	1. Whether the tourists have professional organizations 2. Untimely Rescue 3. Low rescue capacity 4. Poor Rescue Communication 5. Illegal Activities 6. No risk warning information has been released

#### 3.2.2. Classification of influencing factors.

**Fig 4:**
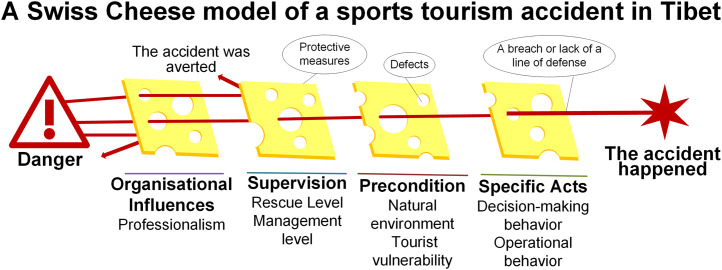
The SCM of sports tourism accidents in the Tibet Autonomous Region of China.

The SCM posits that accidents within an organization are caused by factors at four levels (four slices of cheese), including organisational infuences, supervision, precondition, and specifie acts [18] (Fig 4). Based on the factors extracted from the fishbone diagram, this paper hierarchically classifies 17 factors with the aid of SCM. In the “Organisational Infuences” layer, “organizational professionalism” is summarized. In the “Supervision” layer, “rescue level” and “management level” are summarized. The “Precondition” layer summarizes “natural environment” and “tourist vulnerability”. The “Specifie acts” layer summarizes the “visitor behavior” factors, which are specifically divided into “action class behaviors” and “decision-making behaviors”.

(1) Organisational infuences

A travel team constitutes a temporary organization comprising all participants and a tour leader, where the tour leader holds decision-making authority over the team [53]. At the organizational level, the secondary indicator “organizational professionalism” is established based on the nature of sports tourism groups, indicating the lack of organizational support during activities, informal organizational structures or affiliations, and insufficient professional staffing within the organization.

(2) Supervision

At the management level, two secondary indicators are defined: “rescue capabilities” and “management standards”. Rescue capabilities reflect organizers’ inadequate risk response capacities, including deficient emergency response systems from scenic areas’ authorities or local governments and insufficient post-accident handling due to negligence [54,55]. Management standards highlight insufficient macro-level regulation and oversight of the sports tourism industry by governments. Many sports tourism enterprises—such as travel agencies and scenic areas—exhibit poor operational practices, outdated operational philosophies, and inadequate risk forewarning systems or infrastructure maintenance [34].

(3) Precondition

This tier accounts for Tibet’s unique environmental context through two indicators: “natural environment” and “tourist vulnerability”. Natural environment refers to specific environmental conditions that may harm participants or cause losses [56]. Tourist vulnerability encompasses participants’ physical fitness, sports tourism experience and skills, and susceptibility to altitude sickness [57,58].

(4) Specifie acts

This tier focuses on tourist actions during sports tourism activities, including: inadequate equipment preparation prior to travel; poor decision-making due to herd mentality or weak safety awareness; risk-taking behaviors during activities, such as disregarding the tour leader’s instructions, refusing to cooperate with staff, or improper operational practices [59,60].

### 3.3. Variable design

#### 3.3.1. Conditional variables.

According to the analysis results of the Fishbone diagram of the accident case, the SCM constructed in the previous paper is extended, quantified and operationalized, and the specific condition variables are obtained (Table 3). There are generally three calibration methods for QCA: direct assignment, direct calibration method and indirect calibration method. The assignment of variables in fsQCA is a crucial step, determined by researchers based on their experience and relevant theoretical judgments. This process relies on external knowledge rather than the inherent attributes of the data itself [61]. Therefore, this study makes use of the existing theoretical and empirical evidence, combines the assignment suggestions of relevant experts, adopts the direct assignment method, and strictly follows the principle of “the guidance of theory and/or the relevance of practice” proposed by Ragin [62].

**Table 3:** Condition variables.

Dimension of Variables	Name of Variables	Details	Key references
Organisational Infuences	Organizational professionalism	Tourists join sports tour groups organized by travel agencies or clubs, or the groups have professionals.	Priporas, et al., 2018 [63]; Müller, et al., 2016 [53]
Supervision	Level of rescue	The rescue capabilities of the scenic area authorities or local governments, including equipment, personnel allocation and response time, etc.	Banerjee, et al., 2021 [54]; Wang et al., 2024 [55]
	Management level	The government’s supervision over the sports tourism industry is insufficient, including infrastructure, risk early warning and tourism norms, etc.	Mata and Carvalhinho, 2022 [6]; Koç, 2016 [34]
Precondition	Natural environment	Specific natural environmental conditions that may harm participants or cause losses.	Burtscher, et al., 2018 [64]; Ren, et al., 2020 [65]; Geneletti, 2007 [66]
	Tourist vulnerability	Physical fitness and experience skills that affect tourists’ sensitivity to the altitude of the plateau.	Burtscher, et al., 2018 [64]; Buckley, 2012 [67]; Fluker and Turner, 2000 [68]
Specific Act	Tourist behaviour	The risky behaviors and wrong decisions of tourists in sports tourism activities.	Woodman, et al., 2010 [69]; Giddy and Webb, 2016 [70]

In fsQCA, membership scores range from 0, indicating complete non-membership, to values between 0 and 0.5, signifying fuzzy non-membership. The midpoint, 0.5, represents the crossover point of the result set. Values between 0.5 and 1 indicate fuzzy membership, while a membership score of 1 denotes complete membership [22]. The variables RL, GL, NE, TV, and AB draw on the four-scale Likert scale and are assigned values using the four-valued fuzzy set (0, 0.33, 0.67, 1) [71]. The variable OP is assigned a value using a three-valued fuzzy set, with three score levels of 0, 0.75, and 1 selected to avoid intermediate value situations [72]. The variable DB indicates whether tourists make dangerous decisions and is a clear condition that is essentially dichotomous [73]. Therefore, when the condition exists, it is assigned a value of 1; when it does not exist, it is assigned a value of 0, which avoids information distortion and meaning distortion. To reduce the subjectivity of assignment, this study adopts double-blind and independent assignment. Two researchers respectively assigned values to each accident case based on the accident text. The two coders invited a third researcher to arbitrate the items with inconsistent assignments against the original text. Eventually, a 100% consensus was reached, forming a conditional variable dataset for fsQCA. The specific assignment rules are outlined in Table 4.

**Table 4:** Assignment rules.

Dimension of Variables	Name of Variables		Code of Variables	Details	Assignment Rules
Organisational Infuences	Organizational professionalism		OP	Are tourists organized and is there a professional (certified) guide or leader in the organization	1. Professional organizations is assigned a value of 0.00 2. Non-professional organizations (network recruitment, chance encounters, etc.) is assigned a value of 0.75 3. Unorganized (alone) is assigned a value of 1.00
Supervision	Level of rescue		RL	1. The rescue was not timely 2. Low rescue capability (including number of personnel, equipment, etc.) 3. Poor rescue communication (no signal)	The absence of the appeal situation in the case is assigned a value of 0.00; satisfaction of one condition is assigned a value of 0.33; satisfaction of two conditions is assigned a value of 0.67; satisfaction of all conditions is assigned a value of 1ll three conditions satisfy are assigned a value of 1.00
	Management level		GL	1. Relevant activities without the review of the relevant departments, unregistered, illegal 2. To the scenic spot and the scenic spot peripheral environment management is not good, has not released the risk early warning information and so on 3. Infrastructure is not perfect, not regular inspection and maintenance of related facilities and equipment	The absence of the appeal situation in the case is assigned a value of 0.00; satisfaction of one condition is assigned a value of 0.33; satisfaction of two conditions is assigned a value of 0.67; satisfaction of all conditions is assigned a value of 1ll three conditions satisfy are assigned a value of 1.00
Precondition	Natural environment		NE	1. Atmospheric environmental risk (severe weather or elevation greater than 5000 m) 2. Geo-environmental risk (glaciers, harsh terrain, etc.) 3. Natural disaster	The absence of the appeal situation in the case is assigned a value of 0.00; satisfaction of one condition is assigned a value of 0.33; satisfaction of two conditions is assigned a value of 0.67; satisfaction of all conditions is assigned a value of 1ll three conditions satisfy are assigned a value of 1.00
	Tourist vulnerability		TV	1. Physical vulnerability: weak constitution, elderly population, pre-existing medical history 2. Skills vulnerability: first-time participation and lack of experience with skills 3. Pathological vulnerability: altitude sickness	The absence of the appeal situation in the case is assigned a value of 0.00; satisfaction of one condition is assigned a value of 0.33; satisfaction of two conditions is assigned a value of 0.67; satisfaction of all conditions is assigned a value of 1ll three conditions satisfy are assigned a value of 1.00
Specific Acts	Tourist behaviour	Action class behavior	AB	1. Inadequate preparation 2. Accident due to improper operation (e.g., Slip, fall, etc.) 3. Act of taking risks, e.g., driving too fast, knowingly, disobeying orders, etc	The absence of the appeal situation in the case is assigned a value of 0.00; satisfaction of one condition is assigned a value of 0.33; satisfaction of two conditions is assigned a value of 0.67; satisfaction of all conditions is assigned a value of 1ll three conditions satisfy are assigned a value of 1.00
		Decision-like behavior	DB	Tourists have low safety awareness behaviors such as blind confidence, underestimating risks and making wrong choices	In the case, a score of 0 is assigned when there is no appeal situation, and a score of 1 is assigned when the condition occurs

**Note:** The secondary variable “natural environment” specifically defines “atmospheric environmental risks” as the occurrence of extreme weather conditions or sports tourism destinations situated at altitudes above 5,000 m. The“geological environmental risks” refers to destinations located in glacial or snow-capped mountain areas, or cases where reports explicitly highlight the presence of hazardous terrain conditions at the tourism site.

#### 3.3.2. Outcome variables.

This paper uses the “Interim Provisions on the Implementation of Tourism Safety Management” (1994) and the National Tourism Administration Order No.41, “Tourism Safety Management Measures” (2016), combined with specific contents from case reports, to assign variables for casualties and missing persons in accidents as the result variables for analyzing accident influencing factors. The severity of the casualties is processed using fuzzy values, with the assignment method detailed in Table 5.

**Table 5:** Outcome variables and assignment rules.

Name of Variables	Code of Variables	Details	Assignment of Variables
Severity of the accidents		According to the “Interim measures for the implementation of Tourism Safety Management Rules” (1994) and the selected accident case content, the accident is divided into four grades: minor, general, major and extremely serious accidents	1. Minor accident: Traveler slightly injured or lost contact with not more than 24 hours, assigned a value of 0 2. Ordinary incident: Traveler seriously injured or lost contact for more than 24 hours, assigned a value of 0.33 3. Significant accidents: one tourist killed (including missing) or tourists maimed seriously, assigned a value of 0.67 4. Major accidents: several tourists killed (including missing), or the nature of a particularly serious major impact, assigned a value of 1.00

### 3.4. Configuration path analysis with fsQCA

#### 3.4.1. Analysis of the necessity of individual conditions.

Before conducting the fuzzy-set configurational analysis, it is essential to determine whether a single condition variable serves as a necessary condition for the occurrence of sports tourism safety accidents in Tibet. fsQCA 3.0 was employed to quantitatively analyze the necessity of individual conditions, and the results are presented in Table 6. In severe accidents, the consistency levels of condition variables do not exceed 0.9, indicating that they are not necessary conditions for severe accident occurrence. The analysis shows that no single condition variable is sufficient to cause severe accidents or general accidents, thus necessitating a multivariate combinatory analysis.

**Table 6:** Analysis of the necessity of a single condition.

Conditions		Result			
		Serious accidents (R)		General incidents (~R)	
Dimension of Variables	Code of Variables	Consistency	Coverage	Consistency	Coverage
Organisational Infuences	OP	0.760	0.576	0.856	0.568
	~OP	0.430	0.773	0.362	0.569
Supervision	RL	0.371	0.905	0.265	0.567
	~RL	0.823	0.561	0.956	0.571
	GL	0.468	0.705	0.532	0.703
	~GL	0.803	0.662	0.777	0.561
Precondition	NE	0.686	0.729	0.599	0.559
	~NE	0.585	0.625	0.710	0.665
	TV	0.390	0.605	0.577	0.785
	~TV	0.862	0.699	0.710	0.505
Specific Acts	AB	0.704	0.705	0.755	0.663
	~AB	0.663	0.756	0.664	0.663
	DB	0.726	0.538	0.711	0.462
	~DB	0.274	0.520	0.289	0.480

#### 3.4.2. Analysis of combinations of condition variables.

After completing the necessity analysis, the truth tables corresponding to severe accidents and general accidents are constructed. For the analysis, fsQCA 3.0 was employed, with the consistency threshold set at 0.8, the Proportional Reduction in Inconsistency (PRI) threshold at 0.7, and the case frequency threshold at 1, considering the small sample size (15–40). Cases that do not meet the consistency standard are coded as 0 and excluded [60].

Based on the Boolean minimization rules for configurational paths, the truth tables of the two types of accidents were analyzed to generate three types of solutions: parsimonious, complex, and intermediate solutions. In this study, the intermediate solution is used as the standard configuration result, supplemented by the parsimonious solution. Conditions that appear in both the intermediate and parsimonious solutions are regarded as core conditions, while those appearing only in the intermediate solution are considered peripheral conditions. Core conditions exert a greater influence on the outcome variables than peripheral ones [74].

Using fsQCA 3.0 software, a total of nine configurational paths were identified for the influencing factors of both severe and general accidents, as shown in Table 7.

**Table 7:** Configuration path of influencing factors of Tibet’s sports tourism safety accidents.

Overall, the consistency levels of both individual and overall solutions for severe and general accidents exceed the minimum threshold of 0.7. Specifically, there are three configurational paths for severe accidents with an overall solution consistency of 0.888 and a coverage of 0.425; and six paths for general accidents with an overall solution consistency of 0.876 and a coverage of 0.634. These results indicate that the nine configurations of antecedent conditions exhibit a high degree of consistency in explaining the occurrence of sports tourism safety accidents in Tibet, and can thus be considered sufficient conditions for such accidents.

#### 3.4.3. Robustness check.

To verify the robustness of the configuration conclusion, the sufficiency threshold of the truth table was raised from 0.80 to 0.85. After the threshold was raised, the case frequency threshold remained unchanged at 1, and the overall causal path framework also remained constant. Only minor adjustments were made to the core conditions of the severe accident configurations H1a and H1b: the missing core changed from “low tourist quality” to “low management level”. However, univariate consistency shows that “low tourist quality” (0.390) is still higher than “low management level” (0.371), suggesting that this substitution does not substantially weaken the explanatory power.

What is particularly noteworthy is the change in the overall coverage rate: at the 0.80 threshold, the overall coverage rate is 0.425, while at the 0.85 threshold, it drops to 0.405. This reduction indicates that although raising the consistency threshold enhances the homogeneity within each configuration, it simultaneously excludes some “marginal cases” within the 0.80–0.85 range, thereby narrowing the accident extension that the identified configuration can explain. This indicates that although the threshold of 0.85 is highly applicable to extreme consistency cases, its explanatory power is limited for heterogeneous situations in the consistency transition zone. Therefore, this paper analyzes the configuration path with a consistency threshold of 0.8 and a frequency threshold of 1, and the conclusion obtained has high robustness.

### 3.5. The formation mechanism of sports tourism safety accidents

The fsQCA analysis results show that the occurrence of sports tourism safety accidents on the Qinghai-Tibet Plateau is the result of the combined effect of multiple factors. According to the two categories of serious accidents and general accidents, an in-depth analysis was conducted on the obtained 9 configuration paths.

#### 3.5.1. Mechanism analysis of serious accidents.

The sufficiency analysis of severe accidents generates three causal configuration paths, which are classified into two major categories: natural factor dominated type and human factor dominated type.

(1) Natural Factor Dominated Type

Configuration H1a: In this path, the core factors are the presence of natural environment, the lack of organizational professionalism, and the absence of tourist vulnerability. The auxiliary factors are the lack of rescue level and tourist behavior. This path shows that the special natural environment and sudden natural disasters in Tibet constitute the primary driving factors of the accident. When such force majeure risk sources occur, even if the tourism organization has a high professional level and the tourists’ behavior complies with the norms, the rescue system is difficult to respond quickly due to poor spatial accessibility and limited technical equipment. The disaster impact will still break through the safety threshold with an overwhelming advantage, resulting in serious casualties. The typical case corresponding to this configuration is the avalanche hiking accident in Nyingchi City. This case was a hiking project with a large number of participants and high professionalism. Most of the tourists involved were inexperienced outdoor sports enthusiasts. The route was not fixed and they encountered natural disasters that could not be predicted.

Configuration H1b: In this path, the core factors are the presence of natural environment, the lack of organizational professionalism, and the absence of tourist vulnerability. The auxiliary factors are the presence of tourist behavior and the lack of management level. This path indicates that outdoor sports tourism activities have high environmental requirements. When the static risk at the destination remains persistently high, sports tourism tourists usually have a mentality of taking chances and taking risks. Especially the “expert confidence” of experienced tourists will induce risky and unsafe behaviors, thereby activating potential risks into actual accidents. The typical case corresponding to this configuration is the “Xizang Adventure King” accident of falling into the water. The party involved had many years of experience in glacier exploration, but due to overconfidence in the stability of the ice tongue structure, they chose to take pictures at the edge of the waterfall that had not been surveyed. Eventually, they fell into the glacier due to the instantaneous disintegration of the ice surface.

Configuration H1a and H1b show a high similarity, and both of them jointly verify that the poor natural environment is an important safety hazard of sports tourism in Tibet. However, the difference lies in that both present two typical causative patterns in the tourist behavior subsystem: “fault-free type” and “risky type”. H1a emphasizes the suddenness of disasters, and H1b highlights the “experience paradox” of professional tourists in high-risk scenarios.

(2) Human Factor Dominated Type

Configuration H2: In this path, the core factors are the presence of rescue level and tourist behavior, with the presence of natural environment, the lack of management level, and the absence of tourist vulnerability acting as auxiliary factors. This path indicates that in the context of high-altitude sports tourism, the natural environment itself constitutes a high baseline risk, and the unsafe behaviors of tourists, as a triggering mechanism, transform potential risks into immediate dangers. Therefore, a crucial interaction effect is formed between tourists’ unsafe behaviors and the low level of rescue. The essence of this path is a systematic failure with a triple coupling of “behavior triggering - response failure - environmental amplification”. This configuration corresponds to typical cases of rockfall accidents on the Sichuan-Tibet Highway and a landslide accident during a hiking trip in Nyingchi City.

#### 3.5.2. Mechanism analysis of general accidents.

The sufficiency analysis of general accidents generates six causal configuration paths, which are divided into four main categories: tourist-altitude sickness type, tourist-experience type, tourist-behavior type, and institutional factors dominated type.

(1) Tourist-Altitude Sickness Type

Configuration L1a: In this path, the core factors are the presence of tourist vulnerability and operational behavior, with the presence of organizational professionalism, natural environment, and the absence of rescue level and management level as auxiliary factors. This path indicates that even if the external supervision and rescue system is relatively complete, individual physical fragility and operational errors can still directly trigger altitude sickness related accidents. At this point, the negative effects of the organization and the environment were compressed to a “potential but unintervened” state, and the accident was mainly attributed to the immediate coupling of the tourists’ own physiological threshold breakdowns and behavioral deviations. This configuration corresponds to the typical case of a child suffering from altitude sickness on Mount Sapu.

Configuration L1b: In this path, the core factors are the presence of tourist vulnerability and operational behavior, with the presence of organizational professionalism, natural environment, and decision-making behavior, as well as the absence of rescue level, serving as auxiliary factors. Compared with L1a, this path extends the causal chain from a single operational error to a dual absence of “cognition and operation”. Insufficient information search before travel and distorted risk assessment during the trip constitute the defect of pre-decision-making, while real-time operational errors act as triggers, turning potential high reversal into explicit accidents. This configuration corresponds to the typical cases of heatstroke on the Dongda Mountain cycling trip and a fall during a cycling trip in the Qiangtang Wilderness.

Configuration L1a and L1b exhibit a high degree of similarity, and the two paths jointly reveal the vulnerability of the “individual physiological - behavioral - cognitive” continuum in plateau sports tourism. When tourists with a relatively low threshold of their own physical fitness are simultaneously exposed to the low-oxygen environment at high altitudes and high-intensity exercise loads, operational errors and decision-making flaws will superimpose and amplify the physiological stress response, significantly increasing the probability of general accidents. The lack of professionalism of the organization increases the possibility of unsafe behavior among tourists, and the poor environmental conditions increase the possibility of accidents such as altitude sickness among tourists.

(2) Tourist-Experience Type

In this pathway, the presence of tourist vulnerability and the absence of organizational professionalism play core roles, while the existence of tourist decision-making behaviors and the absence of rescue capabilities, management standards, natural environmental factors, and tourist operational behaviors serve as supplementary factors. This path indicates that although the existence of professional organizations can provide standardized processes and immediate error correction mechanisms at the operational end, it cannot offset the experience gap and cognitive arrogance at the individual level of tourists. Tourists’ lack of prior knowledge about the environmental risks of the plateau and the expansion of their self-efficacy will jointly give rise to “blind confidence” type wrong decisions. Even if the organization has professional qualifications, tourists’ decision-making mistakes can still directly penetrate the organizational barrier and trigger general accidents. Therefore, the essence of this path is that “experiential vulnerability” and “cognitive distortion” are exposed in a localized and case-by-case manner through the protective net of professional organizations. This proves that in high-altitude sports tourism, the quality of tourists’ cognitive decision-making is a key influencing factor for safety outcomes, while the functional boundary of whether an organization is professional only lies at the behavioral execution level. The corrective effect on cognitive dissonance is limited. A representative case is the Mount Wula altitude sickness incident.

(3) Tourist-Behavior Type

Configuration L3a: In this pathway, the presence of tourist operational behaviors and the absence of natural environmental factors and tourist decision-making behaviors serve as core conditions, while the presence of organizational professionalism and the absence of rescue capabilities, management standards, and tourist vulnerability act as supplementary factors. This path indicates that in Tibet, where self-driving and cycling Tours are prevalent, tourists lacking professional and sports tourism experience are prone to accidents due to improper operation by themselves, and non-professional organizations are also unable to provide immediate corrections at the operation end. Therefore, the operational mistakes of tourists may cross the environmental threshold. Even when the natural environment of the sports tourism destination is good, general accidents still occur. A corresponding case is the Ngari No-Man’s Land Off-Road vehicle crash.

Configuration L3b: In this pathway, the presence of tourist operational behaviors and the absence of natural environmental factors and tourist decision-making behaviors remain core conditions, while the presence of organizational professionalism, management standards, tourist vulnerability, and the absence of rescue capabilities serve as supplementary factors. This path indicates that non-professional organizations and low management levels jointly weaken risk identification, hardware preparation and process control. The vulnerability of tourists further reduces the team’s sensitivity to danger signals and self-restraint ability. In this context, operational errors are no longer occasional instantaneous fluctuations but are systematically created under the dual effects of organizational failure and individual insufficiency. A representative case is the Nyingchi Road collapse incident.

Configurations L3a and L3b share high similarity: accidents occur in relatively safe natural environments due to tourist operational errors. However, unlike L1a, L3a involves tourists with higher competence and adequate management standards, emphasizing the accident’s unpredictability and contingency.

(4) Institutional Factors Dominated Type

Configuration L4: In this pathway, the presence of tourist decision-making behaviors and management standards, coupled with the absence of rescue capabilities and tourist operational behaviors, act as core conditions, while the presence of organizational professionalism, natural environmental factors, and the absence of tourist vulnerability serve as supplementary factors. The logical core of this path lies in the triple coupling of “system - cognition - consequence”. When the official governance system experiences a failure of early warning and a gap in emergency response, even if tourists have basic operational skills, they are very likely to make high-risk decisions due to information vacuum, thereby transforming institutional deficiencies into observable general accidents. A corresponding case is the Namtso Lake disorientation incident, where the managers failed to issue early warnings or close the area despite unseasonable snowfall rendering hiking unsafe.

## 4. Discussion

### 4.1. Comparison with previous studies: Addressing research gaps

This study directly addresses three critical gaps identified in the introduction: (1) the scarcity of research on multifactor coupling effects in tourism accidents, (2) the neglect of plateau-specific risk dynamics, and (3) the overreliance on linear methods ill-suited for complex causality [9–12]. Contrary to prior work that isolates factors such as tourist behavior [4] or environmental hazards [5], this research’s fsQCA reveals nine configuration paths (e.g., “Tourist negligence + weak supervision + communication breakdown”). These factors illustrate how latent and active failures nonlinearly converge to trigger accidents. This fundamentally challenges reductionist approaches in tourism safety literature and extends SCM theory by integrating vulnerabilities specific to the plateau (e.g., altitude-amplified equipment failures).

#### 4.1.1. Spatiotemporal characteristics.

Existing studies confirm significant seasonal and regional variations in sports tourism accidents, with accident rates peaking during high tourist seasons due to surges in visitor numbers and activity intensity [75]. The current study corroborates this by identifying May to July as the period with the highest accident occurrence in Tibet. Additionally, the spatial distribution analysis reveals a concentration of accidents in high-altitude regions like Nyingchi City. This aligns with the research conclusion that geographical features (such as steep terrain and hypoxic environment) attract tourists and increase risks [76]. However, the current study extends this by linking the spatiotemporal patterns to high-risk activities such as hiking and cycling, which are particularly prevalent in these regions. Unlike previous studies that primarily focused on seasonal variations, this research also highlights the concentration of accidents in specific high-altitude areas, providing a more nuanced understanding of the spatial distribution of risks [77].

#### 4.1.2. Influencing factors.

The SCM framework used in this study categorizes influencing factors into active failures (direct triggers) and latent failures (systemic weaknesses). This classification builds upon prior research that has examined human, environmental, and material risks [78]. Identifying natural environmental hazards, such as extreme weather, and tourist behavior, as active failures, alongside organizational gaps and regulatory oversight as latent failures, provides a more nuanced understanding of accident causation. This study systematically categorizes these factors within the SCM framework, offering a clearer distinction between direct triggers and systemic weaknesses.

#### 4.1.3. Risk coupling paths.

Unlike studies attributing accidents to single causes (e.g., “human error” [4] or “weather” [76]), this configurational analysis proves that no necessary condition alone suffices for accidents—consistent with Reason’s SCM but previously untested in plateau tourism [18]. For instance, while tourist negligence is prevalent in Path 7, its effects are neutralized by concurrent regulatory gaps (latent failure). This forces a paradigm shift from factor-centric to pathway-centric risk governance.

A key contribution of this study is the identification of nine configuration paths leading to severe and secondary accidents through fsQCA. This approach highlights the nonlinear interplay between systemic deficiencies (latent failures) and situational triggers (active failures). Previous research has often focused on individual factors in isolation, whereas the current study demonstrates that accidents typically result from the coupling of multiple factors. For instance, the configuration paths for severe accidents, such as “environmental hazard + equipment failure + poor rescue coordination,” and secondary accidents, such as “tourist negligence + weak supervision + communication breakdown,” underscore the complexity of accident causation [79]. This finding is consistent with the study which argued that a combination of factors, rather than a single cause, is typically responsible for accidents [80]. The current study’s emphasis on the interaction between organizational, environmental, and behavioral factors provides a more holistic view of risk management. Unlike previous studies that may have overlooked the interdependencies between different factors, this research highlights the importance of considering multiple pathways to accident occurrence.

### 4.2. Implications

#### 4.2.1. Theoretical implications.

First, Theoretical Extension of the SCM: This study innovatively applies the SCM to high-altitude sports tourism safety, expanding its theoretical scope beyond traditional industries (e.g., aviation, healthcare) to address complex environmental and human-factor interactions in extreme tourism contexts. The refined framework integrates multi-layered defense mechanisms (natural, behavioral, managerial, and rescue layers), offering a systematic lens for analyzing nonlinear risk couplings in fragile ecosystems.

Secondly, Methodological Contribution of fsQCA in Tourism Safety Research: By employing fuzzy-set Qualitative Comparative Analysis (fsQCA), this research advances methodological approaches in tourism safety studies. It demonstrates how fsQCA effectively uncovers multifactorial causal configurations driving accidents, addressing limitations of linear regression models in capturing complexity. This approach sets a precedent for small-sample, case-based analyses in understudied high-risk tourism regions.

Thirdly, High-Altitude-Specific Risk Coupling in Sports Tourism: This study pioneers the identification of plateau-environment-driven risk configurations unique to high-altitude sports tourism. By analyzing the interplay of hypoxia, extreme weather, and glacial terrain with tourist behavior, it reveals how altitude-exacerbated vulnerabilities amplify risks in adventure activities like trekking or cycling. These findings fill a critical gap in existing literature, which often overlooks the nonlinear synergies between high-altitude ecosystems and sports tourism dynamics, offering a theoretical foundation for future research on extreme-environment tourism safety.

#### 4.2.2. Practical implications.

First, this study proposes a multi-layered risk prevention system for sports tourism in high-altitude regions. The system includes natural defense, behavioral defense, management defense, and rescue defense layers, each targeting specific risk factors identified in the study. This comprehensive approach provides practical guidance for policymakers, tourism managers, and safety professionals to develop targeted interventions that address the multifaceted nature of sports tourism accidents. Implementing such a system can significantly enhance safety and reduce the incidence of accidents in high-altitude sports tourism destinations.

Secondly, the study highlights the critical role of visitor behavior and decision-making in accident causation. Therefore, enhancing visitor education and awareness through targeted programs and campaigns is essential. These initiatives should focus on educating tourists about the risks associated with high-altitude sports tourism, providing them with necessary skills and knowledge to make informed decisions, and promoting responsible behavior. By improving visitor preparedness and awareness, the likelihood of accidents can be significantly reduced, thereby enhancing overall safety in sports tourism environments.

Lastly, the findings emphasize the importance of robust emergency response and rescue capabilities in mitigating the severity of accidents. This includes upgrading rescue equipment, deploying advanced technologies such as drones and helicopters for rapid response, and establishing comprehensive emergency communication systems. Additionally, forming collaborative rescue alliances involving government agencies, military, and local communities can enhance the efficiency and effectiveness of rescue operations. Strengthening these capabilities is crucial for minimizing the impact of accidents and ensuring the safety of tourists in high-altitude regions.

#### 4.2.3. Implications for other high-altitude contexts.

Although this study focuses on the Tibet Autonomous Region, the integrated SCM-fsQCA framework and identified risk configurations offer transferable insights for similar high-altitude or extreme-environment destinations (e.g., the Andes, Alps, or Himalayas). The core risk mechanisms—such as hypoxia-driven vulnerabilities, nonlinear couplings between environmental hazards and human/organizational factors, and the criticality of rescue coordination—are universally relevant in high-altitude tourism settings. For instance, the “environmental hazard + equipment failure + poor rescue coordination” path (Path 3 in fsQCA results) mirrors accident patterns observed in Alpine mountaineering [2,34]. The proposed four-layer defense system (natural, behavioral, managerial, rescue) can be adapted to other regions by calibrating location-specific factors (e.g., glacial terrain in the Andes or avalanche risks in the Alps). Future studies should validate these findings in diverse geographic contexts to establish a generalized high-altitude safety management paradigm.

### 4.3. Limitations of the study

This study presents four primary limitations. First, the accident cases were predominantly sourced from online media reports (e.g., news portals and government bulletins). Although this approach facilitated extensive case coverage, it may introduce biases inherent in media reporting, such as the underreporting of minor incidents, and result in incomplete event details [77]. This limitation constrains the representativeness of the sample and hinders subtype-specific analyses, particularly regarding activity-specific risk patterns. Second, the influencing factors were identified retrospectively using the SCM framework; however, these factors are subject to dynamic changes as sports tourism evolves, including the introduction of new equipment technologies and regulations. Third, in accordance with the iceberg theory, unreported near-misses and latent hazards, such as gradual environmental degradation, remain unexamined. Lastly, while fuzzy-set qualitative comparative analysis (fsQCA) identifies configurations of factors, it does not account for the dynamic interactions between these factors, such as how tourist behavior adapts to real-time changes in weather conditions.

## 5. Conclusion

### 5.1. Key findings

This study investigates the influencing factors and configuration paths of safety accidents in sports tourism within the Tibet Autonomous Region, utilizing the Safety Critical Model (SCM) and fuzzy-set Qualitative Comparative Analysis (fsQCA).

Key findings reveal distinct spatiotemporal patterns: accidents predominantly occur during peak tourism seasons (May–July) and are concentrated in high-altitude regions such as Nyingchi City. High-risk activities include hiking and cycling, with environmental extremes, inadequate infrastructure, and human errors identified as critical triggers.

Through the SCM framework, six influencing factors are categorized into two tiers: (1) active failures (direct triggers)—natural environmental hazards and tourist vulnerability/behavior; and (2) latent failures (systemic weaknesses)—organizational professionalism, management level, and rescue capability.

The fsQCA results further confirm that accidents arise from the coupling of multiple factors. Nine configuration paths are identified, distinguishing severe accidents from secondary accidents. These configurations highlight the nonlinear interplay between systemic deficiencies (latent failures) and situational triggers (active failures), underscoring the necessity of holistic risk management strategies.

### 5.2. Practical suggestions

**Fig 5:**
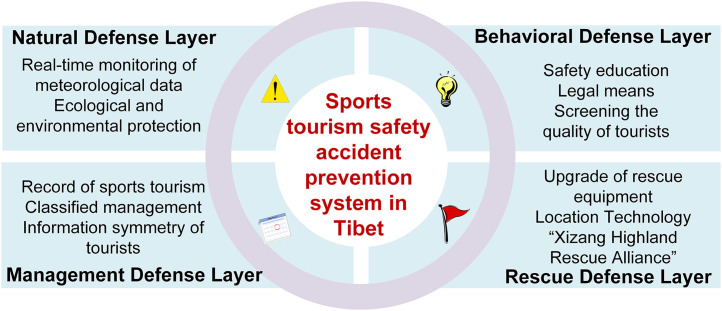
Sports tourism safety accidents prevention system in Tibet.

The analysis results of fsQCA can identify the locations of vulnerabilities in the SCM defense layer, thereby providing data basis for model correction.On the basis of retaining the “potential-active” double-layer failure logic of SCM, this paper makes a directional expansion for the situation of Tibet’s sports tourism, and constructs Tibet’s sports tourism accident prevention system, including the natural defense layer, the behavior defense layer, the management defense layer and the rescue defense layer (Fig 5).The system reproduces and strengthens the “multi-layer and porous” logic of SCM in structure, and makes adjustments to the characteristics of high risk and high consequence of sports tourism in Tibet. At each level, design targeted governance strategies and establish a comprehensive accident prevention and response mechanism to reduce the accident rate and ensure the safety of tourists’ lives and property.

The natural defense layer is an extension of “Precondition”. The configuration analysis results show that the natural environment is not only a static background but can also be “partially shut down” through real-time monitoring and dynamic intervention. Therefore, the natural defense layer transforms “harsh environment” from a passive premise to an active governance target, preventing environmental risks from becoming fixed vulnerabilities that cannot be closed. The behavioral defense layer is a refinement of “Specifie acts”. Combining the influence of tourist behavior and tourist vulnerability factors, tourist behavior is decomposed into experience and skills, risk cognition and behavioral constraints, so that individual mistakes are weakened at multiple levels before they are triggered. The management defense layer isa combination and enhancement of “Organisational Infuences” and “Supervision”. In SCM, organization and management belong to different slices. However, sports tourism in Tibet often presents the characteristics of “temporary teams + mobile supervision” or “traveling alone + ineffective supervision”. Moreover, the root cause of the emergence of unprofessional organizations lies in the inadequate management of the tourism environment. Therefore, this layer combines potential organizational and management deficiencies, transforming the two-layer gaps in SCM into a single layer. The rescue defense layer is a newly established post-event buffer barrier based on SCM. The particularity of high-altitude and high-risk areas such as Tibet makes rescue the last intervening barrier to prevent the escalation of accidents. Therefore, an intervention barrier is added after the original “Specifie acts” of SCM to extend the defense chain.

(1) Natural Defense Layer

Tibet’s unique natural environment increases the inherent risks of sports tourism. To reduce the probability of accidents dominated by natural factors, technological means must be used to narrow the “holes” caused by environmental risks. Establishing a multi-level environmental monitoring and early warning system is one effective way to mitigate safety risks in sports tourism. Through real-time monitoring of meteorological, geological, and hydrological data, natural disaster warnings can be issued in advance, and activity permits restricted during high-risk periods, thereby reducing the likelihood of tourists entering hazardous areas. Additionally, ecological protection should be emphasized during regular tourism development to avoid aggravating environmental fragility.

(2) Behavioral Defense Layer

Tourist-related factors (TQ, AB, DB) are the most frequently occurring actual failure elements across all paths. Preventing this type of accident hinges on reshaping tourists’ risk perception and restraining hazardous behaviors to lower the probability of individual error. First, safety education must be strengthened through scenic area signage, travel agency guidance, and media outreach. Before engaging in sports tourism activities, tourists should be required to complete mandatory training or assessments on subjects such as high-altitude emergency response, fall arrest techniques, and highland travel precautions, thereby enhancing their awareness of natural risks in Tibet. Second, in high-risk environments, people become habituated to hazards they frequently encounter, subsequently underestimating risk and engaging in unsafe behaviors [81]. So tourist behavior must be regulated through strict legal measures. Clear safety signs should be placed in high-risk areas, with undeveloped zones off-limits. Technological tools should be used to monitor real-time tourist behavior in dangerous areas, enabling the timely identification and correction of unsafe actions. Third, for those participating in high-risk sports tourism activities (e.g., high-altitude hiking, cross-country cycling, ice climbing), pre-screening of experience and physical fitness is necessary. Tourists should be required to provide health certificates or experience verification to exclude high-risk individuals in advance.

(3) Management Defense Layer

Safety management of sports tourism should be implemented throughout the entire tourism process, with an emphasis on institutionalization and standardization. Authorities must rigorously review and register high-risk tourism activities, check whether tourists are equipped with necessary safety gear, formulate detailed activity plans and emergency protocols, and enhance the professional level of organizational coordination and safety control [82]. Sports tourism organizations should be classified and managed with clearly defined responsibilities and obligations. The proportion of certified guides in Tibet’s travel agencies should be increased, and it should be mandatory for team leaders to hold “high-altitude dual certification” (Wilderness First Responder and Chinese Mountaineering Association Guide), with regular re-training. Moreover, information asymmetry is a key factor influencing tourists’ poor decisions. Governments must actively monitor content from non-mainstream media that may drive traffic to risky destinations and publish comprehensive and authoritative information on sports tourism through official platforms, including terrain, weather, and route planning, to support informed decision-making by tourists.

(4) Rescue Defense Layer

Rescue system failures amplify the consequences of accidents. A comprehensive and efficient emergency response system should be established to suppress escalation of accident outcomes through rapid intervention. In terms of equipment, relevant rescue facilities should be upgraded. In regions above 5,000 meters in altitude, helicopter standby landing zones should be set up, complemented by unmanned aerial vehicle (UAV) supply delivery systems to ensure arrival at the accident site within one hour and enable precise airdrops of emergency supplies. On the technical level, 5G communication and BeiDou navigation technologies should be used to improve the precision of locating missing persons to within meters, thus shortening response time. Organizationally, a “Tibet Plateau Rescue Alliance” should be established by integrating government, military, and civilian rescue forces. A real-time big data platform should also be developed to synchronize meteorological, topographical, and resource information, optimizing cross-regional coordination and resource allocation.

### 5.3. Further research

First, future research should aim to collect a wider range of accident cases through scientific methods and accurately depict the spatio-temporal differentiation characteristics of sports tourism safety accidents on the Qinghai-Tibet Plateau. Secondly, future research should establish a longitudinal detection framework for long-term monitoring and tracking of accidents. The interactive detection module of the geospatial detector is adopted to explore the interaction mechanism among the influencing factors. Thirdly, various methods such as Markov chains and Bayes are employed to predict the risks of sports tourism on the Qinghai-Tibet Plateau.

## Supporting information

S1 File ~ R Truth table.
(CSV)

S2 File R Truth table. (CSV)

S3 File Original case data. (XLS)

S4 File Supplementary case list. (DOC)

S5 File The Case-by-variable Matrix. (CSV)

S6 File fsQCA results. (TXT)

## References

[1] Wang Y, Lv W, Xue K, Wang S, Zhang L, Hu R, et al. Grassland changes and adaptive management on the Qinghai–Tibetan Plateau. Nat Rev Earth Environ. 2022;3(10):668–83. doi: 10.1038/s43017-022-00330-8

[2] Mairer K, Wille M, Burtscher M. The prevalence of and risk factors for acute mountain sickness in the Eastern and Western Alps. High Alt Med Biol. 2010;11(4):343–8. doi: 10.1089/ham.2010.1039 21190503

[3] Wu K, Wu M, An Z, Jiao H. Evaluation of tourism ecological security and its obstacles in semi-arid river valley urban: a case study of Lanzhou, China. Sci Rep. 2025;15(1):3943. doi: 10.1038/s41598-025-88157-3 39890992 PMC11785809

[4] Plank A. The hidden risk in user-generated content: An investigation of ski tourers’ revealed risk-taking behavior on an online outdoor sports platform. Tour Manag. 2016;55:289–96. doi: 10.1016/j.tourman.2016.02.013

[5] Eitzinger C, Wiedemann P. Risk perceptions in the alpine tourist destination Tyrol—An exploratory analysis of residents’ views. Tour Manag. 2007;28(3):911–6. doi: 10.1016/j.tourman.2006.03.011

[6] Mata C, Pereira C, Carvalhinho L. Safety Measures and Risk Analysis for Outdoor Recreation Technicians and Practitioners: A Systematic Review. Sustainability. 2022;14(6):3332. doi: 10.3390/su14063332

[7] Lepp A, Gibson H. Sensation seeking and tourism: Tourist role, perception of risk and destination choice. Tour Manag. 2008;29(4):740–50. doi: 10.1016/j.tourman.2007.08.002

[8] Lischke V, Berner A, Pietsch U, Mann A. Cardiac Arrest in Mountain Areas during Winter Season Different Causes with Different Out-comes. Notarzt. 2014;30(02):e58–65. doi: 10.1055/s-0033-1360098

[9] Wang M, Wang W, Dai C, Ma C, Luo Y, Xu M. Risk analysis and evaluation of emergency rescue in landslide disaster. Nat Hazards. 2024;120(15):14809–35. doi: 10.1007/s11069-024-06811-x

[10] Khan IU, Motuba D, Vachal K. Investigating factors affecting injury severity of single-vehicle run-off-road crashes. Accid Anal Prev. 2024;208:107786. doi: 10.1016/j.aap.2024.107786 39293190

[11] Eluwole KK, Lasisi TT, Parvez MO, Çobanoğlu C. Application of fuzzy-set qualitative comparative analysis (fsQCA) in hospitality and tourism research: a bibliometric study. J Hosp Tour Insights. 2024;7(5):e3032–54. doi: 10.1108/jhti-08-2023-0572

[12] Vargyas G. Backcountry Skiers, Avalanche Trauma Mortality, and Helmet Use. Wilderness Environ Med. 2016;27(1):181–2. doi: 10.1016/j.wem.2015.09.020 26712332

[13] Ragin CC. The Comparative Method: Moving Beyond Qualitative and Quantitative Strategies. Berkeley: University of California Press; 1987.

[14] Zhou F, Zhang J, Fu C. Generation paths of major production safety accidents - A fuzzy-set qualitative comparative analysis based on Chinese data. Front Public Health. 2023;11:1136640. doi: 10.3389/fpubh.2023.1136640 37033087 PMC10076589

[15] Xuemei L, Wang F, Huili C, Jingwen T. Development of an Indicator System for Evaluating Risks in Trekking Tourism. Leisure Sci. 2024:1–20. doi: 10.1080/01490400.2024.2415941

[16] Lei Y, Zhang G, Lu S, Qian J. Revealing the generation mechanism of cross-regional emergency cooperation during accidents and disasters rescue. Safety Sci. 2023;163:106140. doi: 10.1016/j.ssci.2023.106140

[17] Kumar S, Sahoo S, Lim WM, Kraus S, Bamel U. Fuzzy-set qualitative comparative analysis (fsQCA) in business and management research: A contemporary overview. Technol Forecast Soc Change. 2022;178:121599. doi: 10.1016/j.techfore.2022.121599

[18] Reason J. Managing the Risks of Organizational Accidents. New York: Ashgate Publishing; 2016. doi: 10.4324/9781315543543

[19] Shu-Qin L, Zhen B, Chao-Zong X, Ahmad B, Ming Z, Jian C, et al. Forest biomass carbon pool dynamics in Tibet Autonomous Region of China: Inventory data 1999-2019. PLoS One. 2021;16(5):e0250073. doi: 10.1371/journal.pone.0250073 33939719 PMC8092781

[20] China Tibet TouTiao. The 2024 Tibetan Autonomous Region Sports Industry Exchange conference was held in Nyingchi. 2024 Nov 13. [cited 4 May 2025]. Available from: https://toutiao.xzdw.gov.cn/yw/202411/t20241113_525078.html

[21] China Sports Museum. 2020 China Sports Tourism Quality Project release. 2020 Dec 29. [cited 4 May 2025]. Available from: https://www.olympic.cn/museum/news/benguan/2020/1229/371701.html

[22] Pappas IO, Woodside AG. Fuzzy-set Qualitative Comparative Analysis (fsQCA): Guidelines for research practice in Information Systems and marketing. Int J Info Manag. 2021;58:102310. doi: 10.1016/j.ijinfomgt.2021.102310

[23] Wiegmann DA, Wood LJ, Cohen TN, Shappell SA. Understanding the “Swiss Cheese Model” and Its Application to Patient Safety. J Patient Saf. 2022;18(2):119–23. doi: 10.1097/PTS.0000000000000810 33852542 PMC8514562

[24] Underwood P, Waterson P. Systems thinking, the Swiss Cheese Model and accident analysis: a comparative systemic analysis of the Grayrigg train derailment using the ATSB, AcciMap and STAMP models. Accid Anal Prev. 2014;68:75–94. doi: 10.1016/j.aap.2013.07.027 23973170

[25] Tian S, Wang Y, Li H, Ma T, Mao J, Ma L. Analysis of the causes and safety countermeasures of coal mine accidents: A case study of coal mine accidents in China from 2018 to 2022. Process Safe Environ Protect. 2024;187:864–75. doi: 10.1016/j.psep.2024.04.137

[26] Haghani M, Coughlan M, Crabb B, Dierickx A, Feliciani C, van Gelder R, et al. A roadmap for the future of crowd safety research and practice: Introducing the Swiss Cheese Model of Crowd Safety and the imperative of a Vision Zero target. Safety Sci. 2023;168:106292. doi: 10.1016/j.ssci.2023.106292

[27] da Cunha DT, Hakim MP, Soon JM, Stedefeldt E. Swiss Cheese Model of food safety incidents: Preventing foodborne illness through multiple layers of defence. Food Control. 2022;139:109053. doi: 10.1016/j.foodcont.2022.109053

[28] Hsu W-KK, Shu M-H, Liu Y-C, Wang T-C. Risk Management of Safety for Flight Training in Air Forces. Aerospace. 2022;9(10):558. doi: 10.3390/aerospace9100558

[29] Wu Y, Fu G, Wu Z, Wang Y, Xie X, Han M, et al. A popular systemic accident model in China: Theory and applications of 24Model. Safety Sci. 2023;159:106013. doi: 10.1016/j.ssci.2022.106013

[30] Seshia SS, Bryan Young G, Makhinson M, Smith PA, Stobart K, Croskerry P. Gating the holes in the Swiss cheese (part I): Expanding professor Reason’s model for patient safety. J Eval Clin Pract. 2018;24(1):187–97. doi: 10.1111/jep.12847 29168290 PMC5901035

[31] Akuh R, Atombo C. Road Transport Accident Analysis from A System-Based Accident Analysis Approach Using Swiss Cheese Model. Internat J Eng Ed. 2019;1(2):99–105. doi: 10.14710/ijee.1.2.99-105

[32] Torres Y, Nadeau S, Landau K. Applying AcciMap and STAMP to the analysis of human error in complex manual assembly. Hum Ftrs & Erg Mfg Svc. 2022;32(6):462–81. doi: 10.1002/hfm.20964

[33] Camocini B, Daglio L, Podda R. Sports nature culture. A participatory approach to reconstruct a multidimensional cultural landscape by leveraging outdoor sports. Front Sports Act Living. 2025;7:1554007. doi: 10.3389/fspor.2025.1554007 40125314 PMC11925853

[34] Koc E. Risk and safety management in the leisure, events, tourism and sports industries. Tour Manag. 2016;54:296–7. doi: 10.1016/j.tourman.2015.12.006

[35] Liu J, Wang Z, Li C, Xu R. Exploring pathways to digital transformation: fsQCA analysis based on the AMO framework. PLoS One. 2024;19(12):e0315249. doi: 10.1371/journal.pone.0315249 39729485 PMC11676893

[36] Ragin CC. Redesigning Social Inquiry: Fuzzy Sets and Beyond. Chicago: University of Chicago Press; 2008.

[37] Pu B, Zhou Q, Phau I, Zhu S. Examining the influence of streamer source characteristics on viewers’ continuous viewing intentions in tourism live-streaming: A SEM and fsQCA approach. Asia Pacific J Tour Res. 2025;30(4):437–52. doi: 10.1080/10941665.2025.2454236

[38] Jafari M, Persaud B. Application of a novel hybrid multigroup statistical approach to investigate the factors affecting crash severity. Accid Anal Prev. 2025;214:107985. doi: 10.1016/j.aap.2025.107985 40036922

[39] Callari TC, Bieder C, Kirwan B. What is it like for a middle manager to take safety into account? Practices and challenges. Safety Sci. 2019;113:19–29. doi: 10.1016/j.ssci.2018.10.025

[40] Hock-Doepgen M, Clauss T, Kraus S, Cheng C-F. Knowledge management capabilities and organizational risk-taking for business model innovation in SMEs. J Bus Res. 2021;130:683–97. doi: 10.1016/j.jbusres.2019.12.001

[41] Sun Y, Zheng X, Liu L. Research on generation paths of production safety accidents in urban gas pipeline networks: A fuzzy set qualitative Comparative analysis (fsQCA) based on Chinese data. J Loss Preven Process Ind. 2024;90:105353. doi: 10.1016/j.jlp.2024.105353

[42] Yuxin W, Gui F, Qian L, Jingru W, Yali W, Meng H, et al. Accident case-driven study on the causal modeling and prevention strategies of coal-mine gas-explosion accidents: A systematic analysis of coal-mine accidents in China. Res Policy. 2024;88:104425. doi: 10.1016/j.resourpol.2023.104425

[43] Winge S, Albrechtsen E, Arnesen J. A comparative analysis of safety management and safety performance in twelve construction projects. J Safety Res. 2019;71:139–52. doi: 10.1016/j.jsr.2019.09.015 31862025

[44] Törnberg A, Wahlström M, Lundstedt M, Ekbrand H. Local Conditions for Anti-immigrant Violence: A Qualitative Comparative Analysis (QCA) of Asylum Housing Attacks in Sweden. Terror Polit Violence. 2022;35(6):1353–72. doi: 10.1080/09546553.2022.2042268

[45] Gu J, Wu C, Wu X, He R, Tao J, Ye W, et al. Configurations for positive public behaviors in response to the COVID-19 pandemic: a fuzzy set qualitative comparative analysis. BMC Public Health. 2022;22(1):1692. doi: 10.1186/s12889-022-14097-6 36068522 PMC9449292

[46] Huang R, Xie CW. Configurational analysis of tourism safety accident risk factors in ASEAN countries—based on the HEVP framework and fsQCA. Econ Geography. 2021;41(07):e202–12. doi: 10.15957/j.cnki.jjdl.2021.07.022

[47] Huang R, Xie CW. Characteristics and severity configurations of group outdoor sports accidents. J Beijing Sport Univ. 2022;45(03):e69–83. doi: 10.19582/j.cnki.11-3785/g8.2022.03.008

[48] Huang R, Xie C, Lai F, Li X, Wu G, Phau I. Analysis of the Characteristics and Causes of Night Tourism Accidents in China Based on SNA and QAP Methods. Int J Environ Res Public Health. 2023;20(3):2584. doi: 10.3390/ijerph20032584 36767950 PMC9915894

[49] Senna P, Guimarães Marujo L, dos Santos ACdSG, Freitag AEB, França SLB. Supply chain risk management to achieve healthcare supply chain operational excellence: a fsQCA and PLS-SEM approach. Int J Lean Six Sigma. 2023;15(1):177–200. doi: 10.1108/ijlss-05-2023-0091

[50] Chiu A, Xu Y. Bayesian Rule Set: A Quantitative Alternative to Qualitative Comparative Analysis. J Polit. 2023;85(1):280–95. doi: 10.1086/720791

[51] Ringeval M, Wagner G, Denford J, Paré G, Kitsiou S. Fitbit-Based Interventions for Healthy Lifestyle Outcomes: Systematic Review and Meta-Analysis. J Med Internet Res. 2020;22(10):e23954. doi: 10.2196/23954 33044175 PMC7589007

[52] Chen S, Zhu J. Tourists as commodities in below-cost tours. Ann Tour Res. 2025;110:103890. doi: 10.1016/j.annals.2024.103890

[53] Müller R, Turner JR, Andersen ES, Shao J, Kvalnes Ø. Governance and Ethics in Temporary Organizations: The Mediating Role of Corporate Governance. Project Manag J. 2016;47(6):7–23. doi: 10.1177/875697281604700602

[54] Banerjee I, Warnier M, Brazier FMT, Helbing D. Introducing participatory fairness in emergency communication can support self-organization for survival. Sci Rep. 2021;11(1):7209. doi: 10.1038/s41598-021-86635-y 33785786 PMC8010119

[55] Wang K, Li J, Qiao Y, Chang S. Corporate social responsibility Feng Shui and firm value. Ann Tour Res. 2024;105:103737. doi: 10.1016/j.annals.2024.103737

[56] Petrović MD, Milovanović I, Gajić T, Kholina VN, Vujičić M, Blešić I, et al. The Degree of Environmental Risk and Attractiveness as a Criterion for Visiting a Tourist Destination. Sustainability. 2023;15(19):14215. doi: 10.3390/su151914215

[57] Bentley TA, Page SJ, Macky KA. Adventure tourism and adventure sports injury: the New Zealand experience. Appl Ergon. 2007;38(6):791–6. doi: 10.1016/j.apergo.2006.10.007 17196926

[58] Zeng Y, Filimonau V, Wang L, Zhong L. The impact of perceived unfavorable weather on tourist loyalty in high-altitude destinations: The case of the Qinghai-Tibet plateau, China. J Outdoor Recreat Tour. 2023;43:100658. doi: 10.1016/j.jort.2023.100658

[59] Jeong Y, Kim E, Kim S-K. Understanding Active Sport Tourist Behaviors in Small-Scale Sports Events: Stimulus-Organism-Response Approach. Sustainability. 2020;12(19):8192. doi: 10.3390/su12198192

[60] Li G, Cheng Y, Cai J. Study of risk perception consumption behavior of sports tourism in China. PLoS One. 2023;18(7):e0288735. doi: 10.1371/journal.pone.0288735 37463133 PMC10353816

[61] Wei F, Feng N, Shi B, Evans RD. Collaborative Innovation Performance Within Platform-Based Innovation Ecosystems: Identifying Relational Strategies With fsQCA. IEEE Trans Eng Manage. 2024;71:3496–509. doi: 10.1109/tem.2023.3348154

[62] Ragin CC. Fuzzy-Set Social Science. University of Chicago Press; 2000.

[63] Priporas C-V, Vassiliadis CA, Stylos N, Fotiadis AK. The Effect of Sport Tourists’ Travel Style, Destination and Event Choices, and Motivation on Their Involvement in Small-Scale Sports Events. Event Manag. 2018;22(5):745–65. doi: 10.3727/152599518x15299559637707

[64] Burtscher M, Gatterer H, Burtscher J, Mairbäurl H. Extreme Terrestrial Environments: Life in Thermal Stress and Hypoxia. A Narrative Review. Front Physiol. 2018;9:572. doi: 10.3389/fphys.2018.00572 29867589 PMC5964295

[65] Ren Z, Zuo Y, Ma Y, Zhang M, Smith L, Yang L, et al. The Natural Environmental Factors Influencing the Spatial Distribution of Marathon Event: A Case Study from China. Int J Environ Res Public Health. 2020;17(7):2238. doi: 10.3390/ijerph17072238 32225026 PMC7177444

[66] Geneletti D. Impact assessment of proposed ski areas: A GIS approach integrating biological, physical and landscape indicators. Environ Impact Assess Rev. 2008;28(2–3):116–30. doi: 10.1016/j.eiar.2007.05.011

[67] Buckley R. Rush as a key motivation in skilled adventure tourism: Resolving the risk recreation paradox. Tour Manag. 2012;33(4):961–70. doi: 10.1016/j.tourman.2011.10.002

[68] Fluker MR, Turner LW. Needs, Motivations, and Expectations of a Commercial Whitewater Rafting Experience. J Travel Res. 2000;38(4):380–9. doi: 10.1177/004728750003800406

[69] Woodman T, Hardy L, Barlow M, Le Scanff C. Motives for participation in prolonged engagement high-risk sports: An agentic emotion regulation perspective. Psychol Sport Exerc. 2010;11(5):345–52. doi: 10.1016/j.psychsport.2010.04.002

[70] Giddy JK, Webb NL. The influence of the environment on adventure tourism: from motivations to experiences. Current Iss Tour. 2016;21(18):2132–46. doi: 10.1080/13683500.2016.1245715

[71] Xu J, Cao Y, Wang Y, Qiao Q. Judicial judgment and media sensation of violence against medical staff in China: A fuzzy set qualitative comparative analysis (fsQCA). PLoS One. 2021;16(10):e0259014. doi: 10.1371/journal.pone.0259014 34679107 PMC8535389

[72] Campbell JT, Sirmon DG, Schijven M. Fuzzy Logic and the Market: A Configurational Approach to Investor Perceptions of Acquisition Announcements. Acad Manag J. 2016;59(1):163–87. doi: 10.5465/amj.2013.0663

[73] Zhang X, Zhu H, Hwang Y, Xiao C. Sharing or Not: Psychological Motivations of Brand Rumors Spread and the Stop Solutions. Front Psychol. 2022;13:830002. doi: 10.3389/fpsyg.2022.830002 35444586 PMC9015072

[74] Li P, Bathelt H. Spatial Knowledge Strategies: An Analysis of International Investments Using Fuzzy Set Qualitative Comparative Analysis (fsQCA). Econ Geography. 2021;97(4):366–89. doi: 10.1080/00130095.2021.1941858

[75] Zheng XM, Xie CW, Yin J. Tourism Safety Blue Book: China Tourism Safety Report 2024. Beijing: Social Sciences Academic Press; 2024.

[76] Bitok JJ, Ondigi A, Munyiri E. Foreign Scholars Activities and their Impacts on Sustainable Tourism Development in Nairobi Metropolis, Kenya. J Hospita Tour Manag. 2023;6(1):30–46. doi: 10.53819/81018102t2119

[77] Rosselló J, Sansó A. Yearly, monthly and weekly seasonality of tourism demand: A decomposition analysis. Tour Manag. 2017;60:379–89. doi: 10.1016/j.tourman.2016.12.019

[78] Lai F, Xie C, Zhang J, Huang R. The persuasive effects of warning messages. Ann Tour Res. 2024;109:103829. doi: 10.1016/j.annals.2024.103829

[79] Gonsalves MS, O’Brien B, Twomey DM. Sport and leisure activities in the heat: What safety resources exist? J Sci Med Sport. 2021;24(8):781–6. doi: 10.1016/j.jsams.2021.05.016 34148795

[80] Ge J, Zhang Y, Xu K, Li J, Yao X, Wu C, et al. A new accident causation theory based on systems thinking and its systemic accident analysis method of work systems. Process Safe Environ Protect. 2022;158:644–60. doi: 10.1016/j.psep.2021.12.036

[81] Kim N, Grégoire L, Razavi M, Yan N, Ahn CR, Anderson BA. Virtual accident curb risk habituation in workers by restoring sensory responses to real-world warning. iScience. 2022;26(1):105827. doi: 10.1016/j.isci.2022.105827 36636343 PMC9830218

[82] Szabolcs M, Nagy-Tóth NÁ, Dávid LD, Gogo AFC, Bujdosó Z. The Role of Sports Policing and Tourism Safety at the Summer Olympics. Sustainability. 2022;14(10):5928. doi: 10.3390/su14105928

