# Supplementary material for: Influencing factors of sports tourism safety accidents in Tibet, China: fsQCA analysis based on the SCM
Source: PLoS One. 2025 Oct 27;20(10):e0334226. doi: 10.1371/journal.pone.0334226 (PMC12558482; doi:10.1371/journal.pone.0334226)
Supplement: S4 File — (DOC) [file pone.0334226.s004.doc]

Case Statistics Table

| ID | Location | Time | Content, | Type | Consequence |
| --- | --- | --- | --- | --- | --- |
| 1 | Ali Prefecture | 2020.7 | Exhausted and trapped | Camping | One person was trapped for 23 hours and suffered minor injuries |
| 2 | Nyingchi | 2016.7 | Fall down | Cycling | One person died |
| 3 | Nyingchi | 2019.11 | Get lost and trapped | Hiking | One person got lost for 17 hours |
| 4 | Nyingchi | 2019.5 | Out of contact | Hiking | One person has been out of contact for 50 days |
| 5 | Nyingchi | 2019.4 | Get stuck in the mud | Self-driving | Two people have been out of contact for more than 60 hours |
| 6 | Ali Prefecture | 2024.7 | Slipped and lost contact | Climbing | One person died |
| 7 | Nyingchi | 2017.11 | Out of contact | Climbing | One person died |
| 8 | Changdu | 2018.10 | Slip and fall | Climbing | One person died and one person was injured |
| 9 | Nyingchi | 2014.5 | Slip and fall | Climbing | One person died |
| 10 | Shannan | 2017.1 | Slip and fall | Climbing | One person died |
| 11 | Changdu | 2014.6 | Speeding | Cycling | One person died |
| 12 | Lhasa | 2014.6 | Altitude sickness | Cycling | One person had a mild altitude sickness |
| 13 | Nyingchi | 2023.8 | Altitude sickness | Cycling | One person had a mild altitude sickness |
| 14 | Nyingchi | 2020.12 | Fall into the water by accident | Adventure | One person died |
| 15 | Nagqu | 2017.10 | Out of contact | Hiking | One person died |
| 16 | Ali Prefecture | 2013.5 | Speeding | Cycling | One person was seriously injured |
| 17 | Shigatse | 2013.7. | The landslide was hit by falling rocks | Cycling | One person died |
| 18 | Lhasa | 2010.8 | Fall into the river | Cycling | One person died |
| 19 | Nyingchi | 2016.7 | Sudden hypertension | Cycling | One person died |
| 20 | Shigatse | 2022.2 | The snow-capped mountain is trapped | Hiking | Two people died and three were slightly injured |
| 21 | Ali Prefecture | 2010.6 | The boat capsized while boating on the lake | Cruise ship | Six people died and five were slightly injured |
| 22 | Ali Prefecture | 2012.4 | Slip and fall | Climbing | Two people died, two were seriously injured and three were slightly injured |
| 23 | Nagqu | 2012.10 | Altitude sickness | Hiking | One person died |
| 24 | Lhasa | 2013.5 | Get lost | Hiking | Two people died |
| 25 | Lhasa | 2012.5 | Out of contact | Hiking | One person died |
| 26 | Naqu | 2012.6 | Encounter a mudslide | Climbing | One person died, eight people were trapped for about 24 hours, and 15 people were stranded in the scenic area for 7 days |
| 27 | Naqu | 2019.5 | Out of contact | Hiking | One person died |
| 28 | Ali Prefecture | 2022.7 | Altitude sickness | Climbing | One person suffered from altitude sickness and briefly fainted |
| 29 | Shigatse | 2018.10 | The road surface collapsed and the person was out of contact | Hiking | Two people had mild altitude sickness and were out of contact for 7 days |
| 30 | Nagqu | 2021.11 | Get lost | Climbing | One person has been missing for six hours with minor injuries |
| 31 | Shigatse | 2024.7 | The off-road vehicle lost control | Self-driving | One person was seriously injured and four people were slightly injured |
| 32 | Ali Prefecture | 2017.6 | Car accident | Hiking | One person died |
